# A mid- and long-term follow-up study on the bilateral pedicle anchoring technique with percutaneous vertebroplasty for the treatment of Kümmell's disease

**DOI:** 10.3389/fsurg.2023.1061498

**Published:** 2023-01-26

**Authors:** Shichang Dai, Yu Du, Liang Chen, Yifan Xu, Qiong Hu

**Affiliations:** ^1^ Department of Orthopedic Surgery, The Second Affiliated Hospital of Chongqing Medical University, Chongqing, China; ^2^ Department of Bone and Soft Tissue Oncology, Chongqing University Cancer Hospital, Chongqing, China; ^3^ Department of Radiology, The Second Affiliated Hospital of Chongqing Medical University, Chongqing, China

**Keywords:** Kümmell's disease, percutaneous vertebroplasty, bone cement displacement, pedicle anchoring, stability of bone cement

## Abstract

**Study design:**

Retrospective study of clinical and radiological parameters.

**Objective:**

To investigate the clinical efficacy and long-term stability of bone cement of the bilateral pedicle anchoring technique with percutaneous vertebroplasty (PVP) in the treatment of Kümmell's disease (KD).

**Summary of background data:**

The optimal treatment regimen for KD remains controversial. With the development of minimally invasive orthopedic techniques, PVP has been widely recognized for its advantages, such as less surgical trauma, shorter operation time, less blood loss, quick recovery, and pain relief. Previous reports indicate that in patients who undergo PVP for KD, bone cement may be displaced, causing pain recurrence, or it may enter the spinal canal and cause spinal cord compression, especially in the long term. Theoretically, the bilateral pedicle anchoring technique can enhance the stability of the bone cement in the vertebral body and reduce the occurrence of long-term bone cement displacement. However, there are few reports on the use of this technique to treat KD. This study reports the mid- and long-term follow-up of the clinical and radiological outcomes of the bilateral pedicle anchoring technique with PVP for the treatment of KD.

**Methods:**

From January 2016 to January 2019, 41 patients with KD treated using the bilateral pedicle anchoring technique with PVP in our hospital were enrolled. There were 10 men and 31 women with an average age of 76.5 ± 8.0 years (range: 55–92 years). The average follow-up duration was 19.3 ± 8.0 months (range: 12–38 months). Visual analog scale (VAS) scores, Oswestry disability index (ODI), anterior vertebral height, kyphotic angle, and wedge angle were recorded before surgery, 1 day after surgery, and at the last follow-up. Clinical efficacy, vertebral height recovery, and bone cement displacement were analyzed in combination using plain radiographs, computed tomography, magnetic resonance imaging, and other imaging data.

**Results:**

All the patients successfully underwent the procedure without serious complications. No obvious displacement of bone cement was found in the imaging data obtained 1 day after the operation and at the last follow-up. VAS scores, ODI scores, anterior vertebral height, kyphotic angle, and wedge angle of the injured vertebrae significantly improved after surgery. There was no significant difference between the anterior vertebral height, kyphotic angle, and wedge angle of the vertebral body obtained 1 day after surgery and those obtained at the last follow-up. Bone cement leakage occurred in seven patients, with no abnormal clinical symptoms.

**Conclusion:**

The bilateral pedicle anchoring technique with PVP integrates the use of bone cement in both the vertebral body and the bone cement in the pedicle, enhances the stability of the bone cement, and effectively prevents the displacement of the intravertebral bone cement. The postoperative bone cement stability was high, the clinical effect was obvious, and the long-term follow-up results were satisfactory. Hence, this is a safe and effective surgical method for the treatment of KD.

## Introduction

Kümmell's disease (KD), a complication of osteoporotic vertebral compression fractures (OVCFs), is becoming more common worldwide (1). OVCFs typically respond well to conservative treatment, but KD still affects 7%–37% of the patients (2). KD, also known as delayed osteonecrosis of the vertebral body, has a broad spectrum of clinical manifestations. Therefore, multiple synonymous terms have been used to describe this pathology: posttraumatic vertebral osteonecrosis or avascular necrosis, vertebral pseudarthrosis, intravertebral vacuum cleft or gas, delayed vertebral collapse, and vertebral compression fracture nonunion (1). In addition to pain, intravertebral instability can result from vertebral compression fractures (3). Particularly in KD, intravertebral instability from nonunion of the vertebral fracture can cause persistent severe pain and dysfunction (4). The intravertebral cleft formed through osteonecrosis absorption is an important radiographic feature for diagnosing KD (5). Conservative treatments, such as bed rest and narcotic analgesics, are ineffective in treating patients with KD (6). The relief of back pain, prevention of further collapse of the affected vertebra, and prevention of kyphosis are the objectives of surgical interventions. However, the disadvantages of open surgery, including severe trauma and longer recovery times, are concerning for patients and doctors. Percutaneous vertebroplasty (PVP) is a good treatment option for symptomatic KD (7–10). Although PVP has the potential to alleviate pain, stabilize the vertebral body, and restore vertebral height, it is associated with a greater risk of bone cement leakage and long-term loosening (11, 12). Once bone cement displacement occurs, the vertebral body collapses, and the bone mass at the posterior edge of the vertebral body may enter the spinal canal, cause compression, and induce a series of neurological symptoms (13). We used the bilateral pedicle anchoring technique with PVP to ensure the stability of the bone cement within the vertebral body. We injected bone cement into the vertebral puncture tunnel and pedicle, make bone cement as a whole can get stress support in the pedicle, effectively strengthen the cement in the vertebra fixation. Postoperative follow-up to monitor changes in clinical efficacy-related indicators and explore the surgical treatment of long-term clinical effects of KD was also conducted.

## Materials and methods

### Patient population

This study was approved by the Ethics Committee of The Second Affiliated Hospital of Chongqing Medical University. Written informed consent was obtained for each participant. Forty-one patients were enrolled in this study between January 2016 and January 2019. KD was confirmed in all patients, with segmental lesions at the thoracic (T) or lumbar (L) vertebrae: T7 (2 patients), T8 (3 patients), T9 (2 patients), T10 (1 patient), T11 (6 patients), T12 (7 patients), L1 (11 patients), L2 (4 patients), L3 (4 patients), and L4 (1 patient). Twelve patients had a history of minor trauma, such as trauma from carrying heavy objects or falling on the foot, and the remaining patients had no obvious history of trauma. All patients underwent radiography, computed tomography (CT), and magnetic resonance imaging (MRI). Preoperative bone mineral density measurements of the lumbar spine and femoral neck confirmed osteoporosis. The baseline characteristics of the patients are given in Table 1.

**Table 1:** Baseline characteristics of the patients.

Characteristics of patients (*n* = 41)	
Male/female	10/31
Age (years)	76.5 ± 8.0 (range: 55–92)
Duration of symptoms (months)	3.1 ± 1.4 (range: 1–7)
**Level**	
T7	*n* = 2
T8	*n* = 3
T9	*n* = 2
T10	*n* = 1
T11	*n* = 6
T12	*n* = 7
L1	*n* = 11
L2	*n* = 4
L3	*n* = 4
L4	*n* = 1
Duration of follow-up (months)	19.3 ± 8.0 (range: 12–38)

T, thoracic vertebrae; L, lumbar vertebrae.

### Inclusion and exclusion criteria

The inclusion criteria were as follows: (1) patients with a history of minor trauma or no obvious history of trauma; (2) patients with symptoms lasting more than 3 months or symptoms lasting less than 3 months but with a history of similar symptoms; (3) patients without neurological deficits; (4) patients with CT showing the intravertebral cleft (or vacuum sign) with or without sclerotic margins; (5) patients with a low signal intensity in the cleft on T1-weighted images and either a high or low signal intensity on T2-weighted MRI; (6) patients with osteoporosis; and (7) patients with at least 1 year of follow-up.

Exclusion criteria were as follows: (1) patients with neurological deficit; (2) patients with a history of thoracolumbar surgery; (3) patients with infective lesions on the puncture path; (4) patients with underlying malignancy; (5) patients with serious physical illnesses, abnormal blood coagulation function, or mental disorder; and (6) patients who were unable to tolerate the PVP procedure under local anesthesia.

### Surgical procedures

**Figure 1:**
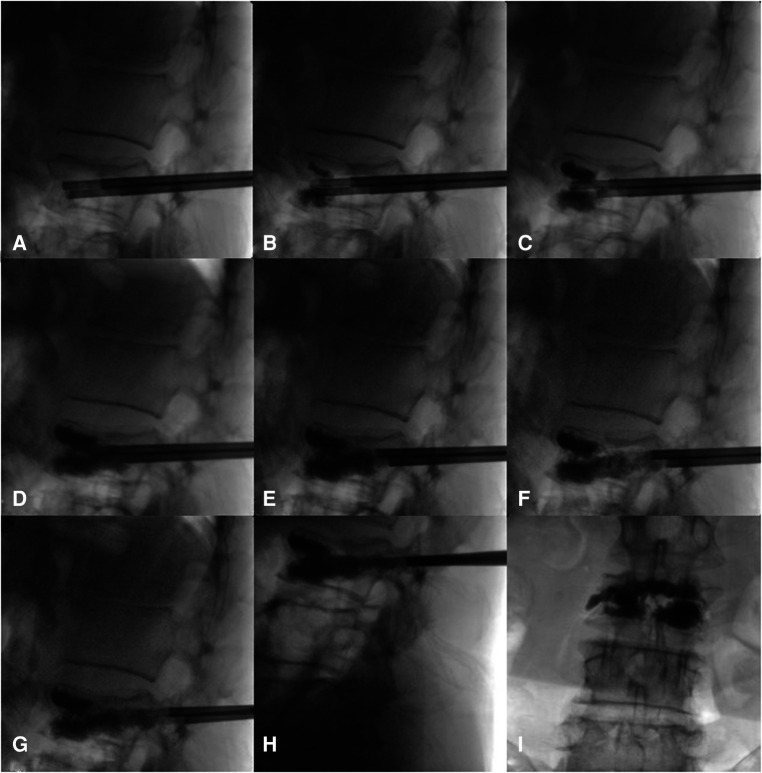
(**A–I**) The intraoperative process of bone cement injection and diffusion.

**Figure 2:**
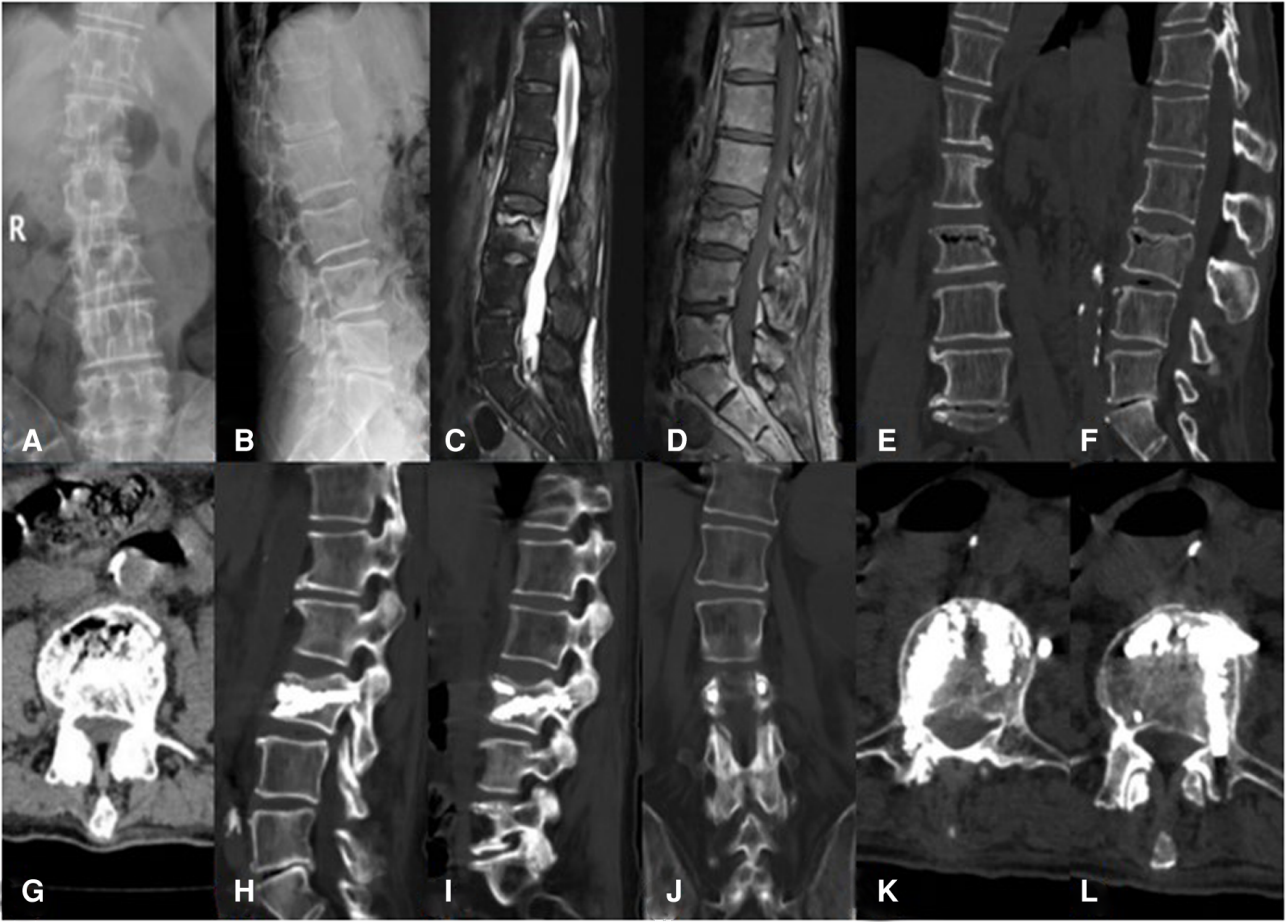
An 81-year-old male patient with KD at L3 treated using bilateral pedicle anchoring technique with PVP: (**A,B**) preoperative radiographic images; (**C**) the sagittal T2-weighted MR image showing high signal intensity in the cleft; (**D**) the sagittal T1-weighted MR image showing low signal intensity in the cleft; (**E–G**) CT scans showing the intravertebral vacuum sign; (**H,I**) CT scans after operation showing the bone-cement-filled cleft and vertebrae; (**J–L**) bone cement dispersion was observed in both pedicles. KD, Kümmell's disease; PVP, percutaneous vertebroplasty.

**Figure 3:**
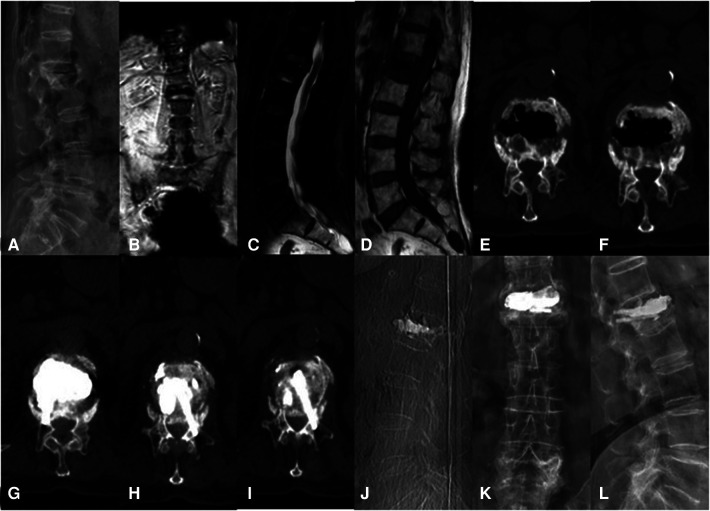
A 76-year-old woman with KD at L1 treated using the bilateral pedicle anchoring technique with PVP: (**A**) preoperative radiographic images; (**B,C**) coronal and sagittal T2-weighted MR image showing low signal intensity at L1; (**D**) the sagittal T1-weighted MR image showing low signal intensity at the same location; (**E,F**) CT scans showing the intravertebral vacuum sign; (**G–J**) CT scans after operation showing the bone-cement-filled cleft and vertebrae; bone cement dispersion was observed in both pedicles; (**J–L**) x-ray at 14 months after operation showing no delayed displacement of bone cement or adjacent vertebral fracture. KD, Kümmell's disease; PVP, percutaneous vertebroplasty.

The surgery was performed under local anesthesia, and the neurological symptoms of the patients were observed in real time. If the patient had any discomfort, they could communicate with the surgeon, who might stop the operation on time and actively resolve the problem. The patient was placed in the prone position; the chest and iliac area were properly cushioned to place them in a slightly extended position; the towel was sterilized; the preoperative and positioning preparations were made; and the projection points of the pedicle surface of both pedicles of the surgical segment were marked. During the operation, the C-arm x-ray machine was used to locate the affected vertebral body and mark the needle insertion points on both sides. After local anesthesia took effect, bilateral pedicle puncture was performed. Based on the preoperative imaging data and x-ray positioning, a point at approximately 10 o'clock on the left pedicle and approximately 2 o'clock on the right pedicle of the injured vertebra, 3–5 mm laterally and at a slightly vertical angle, was selected as the puncture point (the puncture needle point and angle were the same as those of conventional PVP). Under C-arm x-ray machine guidance, the needle channel was adjusted so that it entered from the pedicle and approximately one-third of the needle was inserted proximal to the vertebral fissure. Bone tissue was collected for pathological examination as needed. Bone cement was prepared at the early stage of the wire drawing period, and injected into the center of the vertebral body at the same time under fluoroscopic guidance so that it was evenly distributed in the anterior part of the vertebral body in the form of a mass (the target bone cement could be in the vertebral body fissure and normal bone). After upper and lower support and good distribution of the bone cement were achieved in the anterior and middle columns, the puncture working cannula was retracted to 1 cm from the posterior edge of the vertebral body, and the push rod was injected from the scale of 1 cm and retracted at the same time. A small amount (<0.3 ml each time) of bone cement was injected at the postdrawing stage until the scale was 0 cm; then, this procedure was repeated. Under the monitoring of the anteroposterior and lateral positions, the puncture working cannula was retracted 1 cm from the middle and posterior parts of the pedicle, and the push rod was injected from the scale of 1 cm. At the same time, a small amount (<0.2 ml each time) of bone cement was injected in the late drawing stage or early stage of the globular stage, until the scale was 0 cm, the push rod was rotated, and an empty push rod was used to block the working sleeve to reduce the leakage behind the bone cement. The process of bone cement injection and intraoperative diffusion is shown in Figure 1. Communication with the patients during the operation confirmed that they had no symptoms of nerve irritation, and the large mass of bone cement in front of the vertebral fissure, the bone cement in both puncture passages, and the bone cement in the pedicle were connected to form a triangular overall structure. After the bone cement solidified, the working cannula was pulled out and sterilized and the puncture port was sutured. Typical patients are shown in Figures 2, 3.

### Efficacy evaluation

The visual analog scale (VAS) scores, Oswestry disability index (ODI) scores, anterior vertebral height, kyphotic angle, and wedge angle were recorded before operation, 1 day after the operation, and at the last follow-up to evaluate the effectiveness of the bilateral pedicle anchoring technique with PVP treatment in patients with KD. VAS and ODI analyses were performed using outpatient reexamination or telephone follow-up 1 day after the operation and at the last follow-up. Anterior vertebral height, kyphotic angle, and wedge angle data were collected radiographically before surgery, 1 day after operation, and at the last follow-up. Clinical efficacy, vertebral height recovery, and bone cement displacement were analyzed in combination using preoperative and postoperative plain radiographs, CT, MRI, and other imaging data.

### Statistical analysis

Data were statistically analyzed using the SPSS software (version 26.0; IBM, United States). Normally distributed continuous data were expressed as mean ± SD (x ± s), and the comparison of data at different time points before and after treatment was carried out using paired *t*-test; *p* < 0.05 was considered statistically significant.

## Results

**Figure 4:**
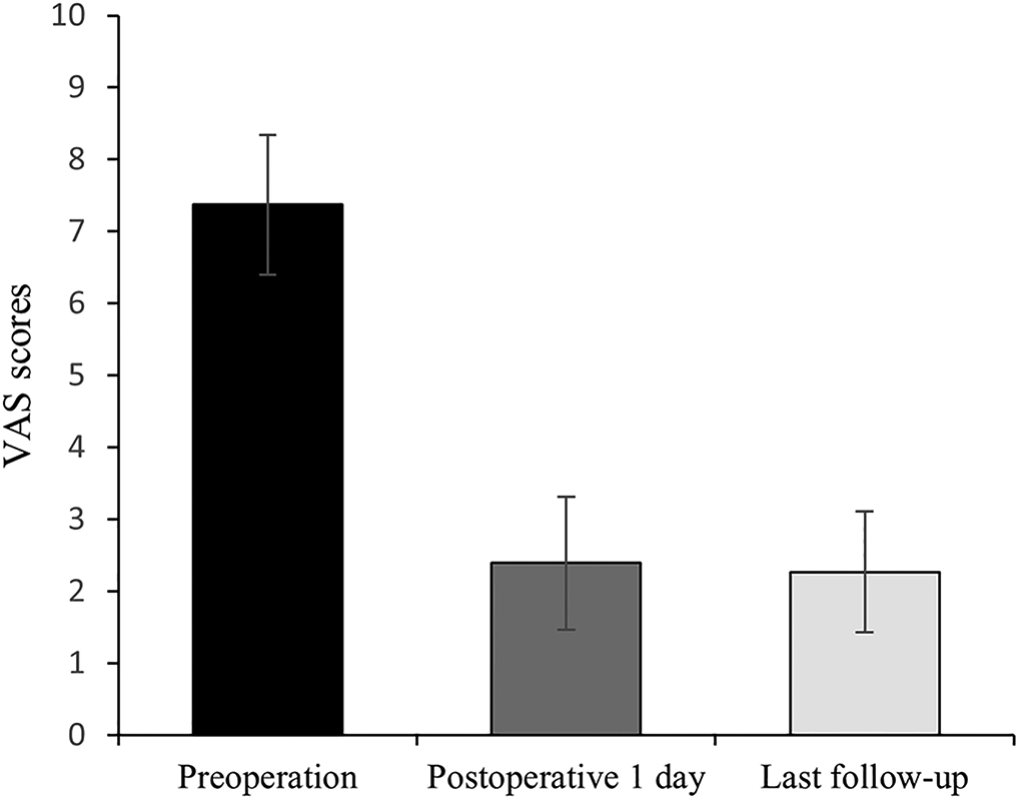
Histograms for VAS scores (n = 41).

**Figure 5:**
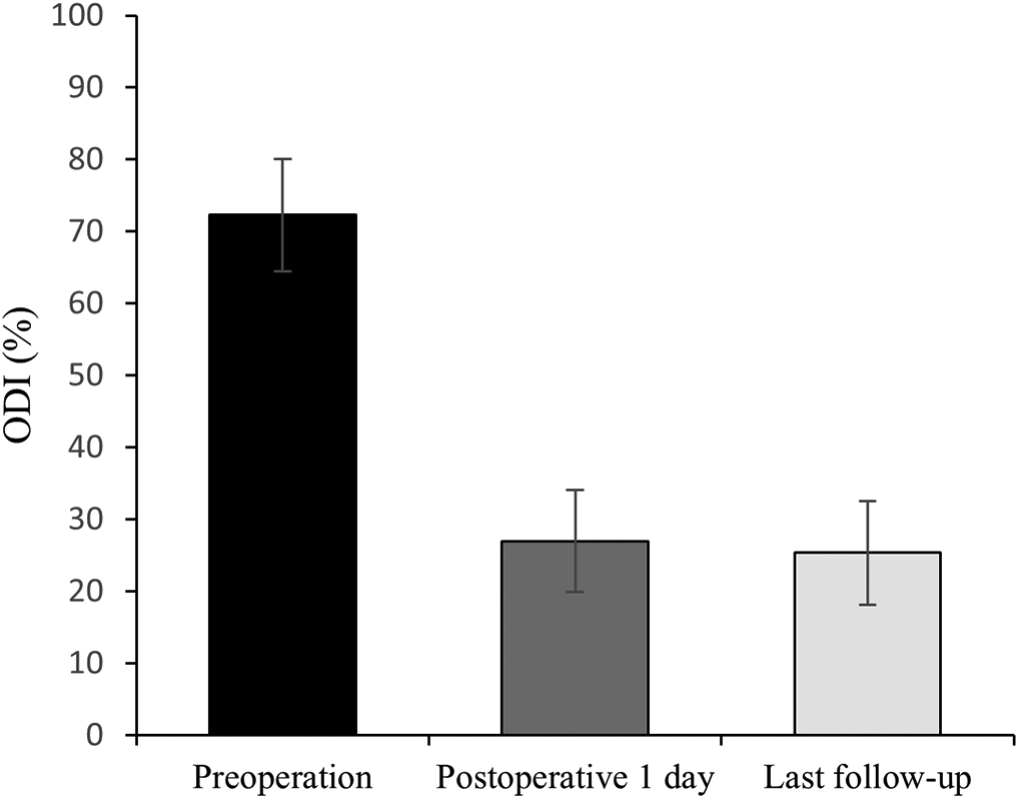
Histograms for ODI (n = 41).

**Figure 6:**
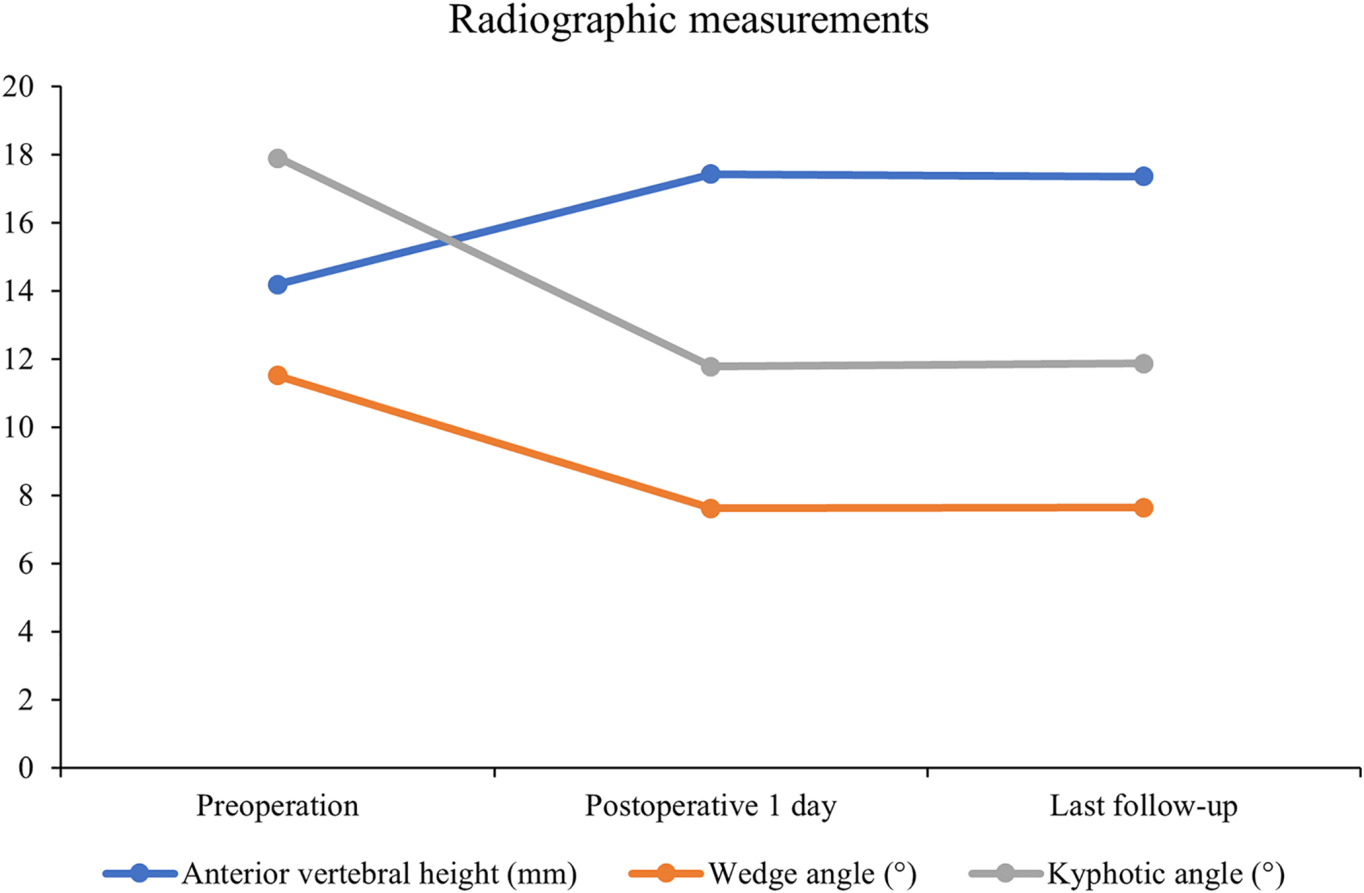
Line charts for radiographic measurements (n = 41).

All 41 patients with KD successfully underwent PVP with the bilateral pedicle anchoring technique. The mean operation time was 38 ± 11 min. The follow-up period of all patients ranged from 12 to 38 months (mean 19.3 ± 8.0 months). The VAS score reduced from 7.37 ± 0.97 preoperatively to 2.39 ± 0.92 and 2.27 ± 0.84 1 day after surgery and at the last follow-up, respectively. The ODI score also decreased from 72.27 ± 7.76 preoperatively to 26.96 ± 7.11 and 25.34 ± 7.23 1 day after operation and at the last follow-up, respectively. Significant statistical differences were observed in both VAS and ODI scores at each time point of follow-up when compared with the preoperative condition (*P* < 0.05, Table 2, Figures 4, 5). In addition, statistically significant improvements in radiographic measurements, such as anterior vertebral height, kyphotic angle, and wedge angle of the involved vertebral body between the preoperative and postoperative assessments, were also observed (*P* < 0.05, Table 3, Figure 6). There was no significant difference in the anterior vertebral height, kyphotic angle, and wedge angle of the vertebral body 1 day after surgery and at the last follow-up (*P* > 0.05). Cement leakage was assessed using immediate postoperative radiography and CT scan. Leakage of bone cement occurred in 7 of the 41 patients (17.07%), including 3 patients of paravertebral soft tissue leakage and 4 patients of intradiscal leakage, and no leakage was found in the spinal canal. Furthermore, no surgical complications, such as neurological deficit, pulmonary embolism, thermal injury, infection, delayed displacement of bone cement, or adjacent vertebral fractures, were observed. No obvious displacement or deformation of the bone cement was observed at the last follow-up compared to 1 day after the operation.

**Table 2:** The VAS and ODI score preoperatively and at each time point postoperatively (*n* = 41).

	Preoperation	Postoperative 1 day	Last follow-up
VAS	7.37 ± 0.97	2.39 ± 0.92	2.27 ± 0.84
ODI	72.27 ± 7.76	26.96 ± 7.11	25.34 ± 7.23

VAS, visual analog scale; ODI, Oswestry disability index.

∗P < 0.05, score at each time point postoperatively vs. preoperative score. Last follow-up occurred at 19.3 months on average, ranging from 12 to 38 months.

**Table 3:** Radiographic measurements of anterior vertebral height, wedge angle, and kyphotic angle (*n* = 41).

	Preoperation	Postoperative 1 day	Last follow-up
Anterior vertebral height (mm)	14.19 ± 3.89	17.42 ± 3.78	17.35 ± 3.81
Wedge angle (°)	11.51 ± 4.90	7.62 ± 3.32	7.65 ± 3.32
Kyphotic angle (°)	17.91 ± 6.15	11.77 ± 5.00	11.87 ± 4.92

∗P < 0.05, score at each time point postoperatively vs. preoperative score. Last follow-up occurred at 19.3 months on average, ranging from 12 to 38 months.

## Discussion

KD is a delayed complication of OVCFs and was first described by Hermann Kümmell in 1895 (14,15). To date, the pathogenesis remains controversial, and two theories have been proposed in the literature. According to the first theory, vertebral osteonecrosis creates the intravertebral vacuum cleft (6, 16), and the second theory indicates that vertebral nonunion and pseudoarthrosis are responsible for the intravertebral vacuum cleft (17, 18). It is possible that a number of factors combine to cause KD. An intravertebral cleft precipitated by trauma is considered the most dominant feature in diagnosing KD, with an incidence rate of 79% (19–21). As a vertebral fracture occurs, gas enters the subchondral cleft and produces a specific gas phenomenon, emitting a lower intravertebral signal visible on MRI (22). Extensive fluid builds up in the cleft over a short period of time as a result of avascular necrosis of the vertebral bodies, which causes intravertebral high signal intensity visible on T2-weighted MRI (23, 24). The cleft may lead to vertebral collapse and spinal canal stenosis, aggravating clinical symptoms (14). At present, surgical treatment of KD is the consensus, and surgery generally does not need to overemphasize the recovery of vertebral height (25), and mainly focuses on pain relief. With the development of minimally invasive technology in orthopedics, PVP and percutaneous kyphoplasty (PKP) are the main surgical methods for treating KD without spinal cord or nerve compression symptoms (26) and have been widely recognized for their advantages, such as less surgical trauma, short operation time, less blood loss, fast recovery, effective recovery of the injured vertebral height, and pain relief (27, 28). However, solving the complication of bone cement displacement and enhancing the stability of bone cement in the vertebral body are difficult problems. Bone cement displacement may occur due to vertebral fissure formation from bone nonunion, the surrounding sclerotic bone disrupting the anchoring of bone cement in the vertebral body, and the incomplete anterior edge of the vertebral body providing the path and space for bone cement displacement and falling off. In recent years, many methods have been proposed to reduce the incidence of bone cement leakage and displacement and increase stability and reliability. Hoppe et al. (29) used the fractional perfusion method to effectively reduce the leakage rate of bone cement. Park et al. (30) used short-segment percutaneous nail placement combined with PVP to effectively stabilize injured vertebrae. Piao et al. (31) used less bone cement and a lower radiation dose to achieve good biological strength of injured vertebrae through unilateral percutaneous bone capsule filling and enhanced vertebroplasty, and they reduced bone cement leakage. Some teams have also used high-viscosity bone cement to strengthen the injured vertebrae and effectively reduce the leakage rate of bone cement (32). PVP with a bilateral pedicle anchoring technique has been used in the treatment of KD at our hospital. The advantages of this technique are as follows: (1) the operation is performed under local anesthesia, the patient is awake, and any discomfort can be timely communicated with the surgeon in a timely manner for adjustment; (2) bilateral perforating cement perfusion can make the bone cement evenly spread in the trabecular bone and vertebral fissure, which improves the stability and support of the injured vertebra; (3) bone cement can form the entire injured vertebra and the pedicle, which further enhances the stability. The VAS, ODI, vertebral anterior height, wedge angle, and kyphotic angle before and after the operation were compared to determine whether the operation was effective, whether it could relieve the pain and discomfort of the patient, and the situation of vertebral compression and collapse. From the results of this study, the relevant indicators have been significantly improved, which proves that the operation can effectively improve the patient's symptoms, improve the quality of life, and stabilize the collapsed vertebral body. In this procedure, the bone cement in the vertebral body was made into a complete whole by bilateral puncture, and then the bone cement was anchored by pedicle shaping of bone cement, so that the bone cement formed a stable structure like a triangle in the vertebral body. The stable distribution of bone cement in the vertebral body was confirmed by clinical and imaging follow-ups. These findings also support the efficacy and advantages of PVP with bilateral pedicle anchoring in the treatment of KD.

This is closely related to the characteristics and advantages of PVP. First, bone cement can be used to fill cracks to stabilize the spine (14), and the high temperature during condensation can burn nerve ends in the vertebral body, thereby relieving pain (33). Second, compared with unilateral puncture, bilateral puncture has slightly lesser requirements for puncture angle, is a relatively easy operation, and achieves a more uniform distribution of bone cement. Compared with the traditional PVP surgery, there are some areas that necessarily require special attention during the operation, mainly focusing on the pedicle anchoring process: (1) avoid selecting patients with incomplete pedicle cortex, so as to reduce the risk of bone cement leakage during intraoperative pedicle formation. (2) In the process of pedicle formation, the bone cement selected should be in the late drawing stage or early dough stage. (3) Standard fluoroscopy should be adjusted during the operation, especially in lateral fluoroscopy; the distribution of bone cement should be even throughout the pedicle. Once the distribution is nonuniform, the operation should be suspended. (4) Standardized use of zoledronic acid, calcitriol, and other antiosteoporosis treatments for patients postoperatively, especially zoledronic acid, can inhibit the activity of osteoclasts and reduce bone conversion and absorption, which can significantly improve osteoporosis in patients (34).

In our study, the postoperative VAS and ODI scores of the patients were lower than the preoperative scores (*P* < 0.05). Postoperative anterior vertebral height, wedge angle, and kyphotic angle significantly (*P* < 0.05) improved compared to preoperative values. The results of the long-term follow-up showed that the imaging parameters did not change significantly compared to 1 day after the operation. We believe that the operation is dependent on the PVP; through bilateral vertebral puncture, the bone cement is more stable, and the distribution is even more stable. It can effectively enhance the fixation and supporting effect of bone cement in the vertebral body, reduce the risk of bone cement displacement, improve the patient's symptoms, and improve the quality of life. This is a safe and effective surgical method for the treatment of KD.

Our study had certain limitations. First, this study lacked direct proof of biomechanics *in vitro* and *in vivo*. Second, the duration of follow-up varied, which might have effected our results. Third, the distribution of injured vertebral segments was relatively large. Differences in the effects of treatment for different vertebral segments were not analyzed. Finally, as the goal of PVP is mainly to strengthen the collapsed vertebrae without aiming for neurological decompression, this minimally invasive technique is not a preferred option in patients with neurological deficits.

## Conclusion

The bilateral pedicle anchoring technique with PVP achieved satisfactory clinical and radiological outcomes, and we believe that this minimally invasive procedure could provide an effective and safe alternative for the treatment of patients with KD. We hope to draw more convincing conclusions with a larger sample size and a longer follow-up duration in the future.

## Data Availability

The raw data supporting the conclusions of this article will be made available by the authors, without undue reservation.
